# Risk assessment of holmium laser induced ureteral stricture: *in-vivo* experiments using a pig model

**DOI:** 10.7717/peerj.21015

**Published:** 2026-03-25

**Authors:** Qiushi He, Qingfeng Huang, Qiao Qi, Yuexian Xu, Bingbing Hou, Zongyao Hao

**Affiliations:** 1 Department of Urology, First Affiliated Hospital of Anhui Medical University, Hefei, Anhui, China; 2 Anhui Provincial Key Laboratory of Urological and Andrological Diseases Research and Medical Transformation, Anhui Medical University, Hefei, Anhui, China; 3 Institute of Urology, Anhui Medical University, Hefei, Anhui, China

**Keywords:** Holmium laser, Ureteral stricture, Porcine model, Thermal dose (CEM43), Macrophage polarization

## Abstract

**Purpose:**

To systematically evaluate the safety boundaries of holmium laser application during ureteroscopy by quantifying thermal dose and to explore the detailed repair mechanisms of the ureter and kidneys following injury.

**Methods:**

Twelve female piglets were selected. Key variables included holmium laser power, irrigation flow rates (0, 7.5, 15 mL min^−1^), and fluid temperatures. Thermal dose was calculated as Cumulative Equivalent Minutes at 43 °C (CEM43). Trauma and repair processes were assessed via microscopic imaging, Masson staining, and immunohistochemistry for inflammatory and fibrosis markers. Statistical analyses were performed using two-way analysis of variance (ANOVA).

**Results:**

At an irrigation flow rate of 15 mL min^−1^, the temperature rise was minimal with a negligible thermal dose (CEM43 < 1), ensuring safety even at 30 W. In contrast, compromised irrigation (7.5 mL min^−1^) or no-flow conditions resulted in rapid heat accumulation, with CEM43 values reaching extreme levels (>10^13^) at high powers. In the kidneys, the repair process involved a transition from inflammation to fibrosis over time, which was correlated with an M1-to-M2 macrophage polarization. Crucially, anatomical ureteral stricture was observed only when the laser-induced injury involved ≥3/4 of the ureteral circumference.

**Conclusion:**

Sufficient irrigation is critical to maintain thermal safety. While thermal exposure induces fibrotic changes driven by macrophage polarization, mechanical injury extent (≥3/4 circumference) appears to be the dominant predictor for the formation of ureteral stricture. These findings emphasize the importance of maintaining high-flow irrigation and minimizing extensive circumferential damage during surgery.

## Background

In recent years, the incidence of urinary stones has remained high, and the disease may have a series of serious consequences, such as leading to ureteral obstruction, frequent urinary tract infections, vomiting or pain, permanent functional damage to the kidneys, and even leading to the development of uremia. Urinary stones have become a global public health problem (Tan et al., 2024). Treatment modes for urinary stones have evolved rapidly over the past two decades. This is due to the proportion of ureteroscopic and ureteral flexible lithotripsy rising as endoluminal urologic techniques continue to mature. Compared with percutaneous nephrolithotripsy, ureteroscopic lithotripsy can also be used for the most stones. It does not show a significant disadvantage in lithotripsy efficiency (Yanaral et al., 2019; Tokas et al., 2021). More importantly, patients are at a lower risk of postoperative complications, less trauma, and less pain. Ureteroscopic lithotripsy is favored by more clinicians and patients.

Ureteroscopic procedures are typically performed by fragmenting or pulverizing the stone with a holmium laser and then removing the stone through a mesh basket or negative pressure suction device. Although the holmium laser has been proven to be a safe and reliable method of lithotripsy (Juliebo-Jones et al., 2023), iatrogenic ureteral stricture still occurs from time to time. Ureteral stenosis further contributes to hydronephrosis, leading to scarring of the renal parenchyma and even inducing permanent decline in renal function (Inoue et al., 2024; Gild et al., 2018).

It has been suggested that such injuries may be related to the thermal effects of the holmium laser, and that even if the holmium laser is not directly excited on the ureteral wall, the increase in lavage fluid temperature can be an important factor in causing ureteral stenosis due to the potential for damage to the uroepithelium and renal parenchymal cells (Tonyali et al., 2023a; Tonyali et al., 2023b). This viewpoint lacks sufficient evidence, and relevant research is limited to *in vitro* experiments, with limited simulation of surgical processes, and cannot demonstrate the trauma and repair process that progresses over time after ureteral thermal injury, which should be questioned (Noureldin et al., 2021). This experiment aims to evaluate the safety and risks of holmium laser during ureteroscopy by simulating the surgical process in live piglets. It also aims to provide a detailed explanation of the trauma and repair process of the ureter and kidneys.

## Methods

All animal experiments were approved by the Animal Experimentation Committee and reviewed by the Ethics Committee of Anhui Medical University (Approval No.: LLSC20242248), and were conducted in strict compliance with national regulations on experimental animal management and ethical standards for animal experimentation.

### Animal model establishment

A total of 12 healthy female piglets (2–3 months old, weighing 35–50 kg) were included in this study. Female piglets were selected to facilitate transurethral ureteroscopic access due to their anatomical suitability, minimizing urethral trauma during instrument insertion. The piglets were donated by Dingyuan County Green Family Breeding Professional Cooperative and acclimated to the experimental environment in the Experimental Animal Center of Anhui Medical University for 2 weeks prior to the experiment to minimize environmental stress. During the acclimation and experimental periods, the piglets were housed under standardized conditions with free access to a standard diet and clean drinking water; the housing density was set appropriately to ensure sufficient activity space for each piglet, meeting the requirements of experimental animal welfare standards. The sample size was determined based on the ethical principle of Reduction (3Rs) and previous experience with similar *in vivo* porcine models (Kose et al., 2024), rather than a formal *a priori* power calculation. The number of animals (*n* = 12) was deemed the minimum necessary to assess safety endpoints and observe significant biological differences in thermal injury markers. Piglets were randomly assigned to groups using a random number generator. For anesthesia, propofol (10 mg/mL) was used, with an induction dose of 0.5 mL/kg administered intravenously *via* the ear margin; anesthesia onset was confirmed by the disappearance of eyelid reflexes, muscle relaxation, and weakened locomotion, and a microinfusion pump was used to maintain anesthesia during the procedure. After the experiment, piglets were euthanized on the day of surgery or 14 days postoperatively *via* anesthesia followed by aortic exsanguination, a humane method that ensures rapid and painless death. Premature euthanasia criteria were established as follows: weight loss of 20–25% or appearance of consumptive symptoms, complete loss of appetite for 24 h or reduced appetite (less than 50% of normal intake) for 3 consecutive days, inability to eat/drink or stand for 24 h without anesthesia/sedation, lethargy with hypothermia (below 37 °C) without anesthesia/sedation, persistent infection unresponsive to antibiotics with systemic discomfort, or intolerable organ lesions; no piglets met these criteria during the study, and all were euthanized as planned. No piglets survived after the experiment; carcasses were handled in accordance with the regulations of the Experimental Animal Center to ensure environmental safety and ethical compliance. Postoperatively, all piglets received daily intramuscular injections of penicillin (40,000 U/kg) for infection prevention. For analgesia, a single dose of buprenorphine (0.01 mg/kg) was administered intramuscularly immediately upon recovery from anesthesia. Adhering to the “Refinement” principle of the 3Rs, we prioritized animal welfare while minimizing experimental interference. To prevent the potential anti-inflammatory effects of continuous analgesics from confounding the histological results, routine postoperative analgesia was limited to the initial dose. Instead, safety was guaranteed through strict humane endpoints, defined as: (1) a Pig Grimace Scale (PGS) score indicative of moderate-to-severe pain, (2) inability to stand or ambulate, or (3) complete loss of appetite for >24 h. Animals were monitored every 4 h, and any subject meeting these endpoints would have immediately received rescue analgesia or euthanasia. During the study, no animals reached these thresholds.

### Experimental projects and operational procedures

For the first experimental project focusing on the effect of lavage fluid flow rate and holmium laser power on temperature rise, after anesthetizing the piglets, a skin-nephro channel was established under ultrasound guidance, and a ureteroscope was inserted through the urethra. Holmium lasers of different powers were activated under different lavage fluid flow rates (15 mL min^−1^, 7.5 mL min^−1^, 0 mL min^−1^), and a sterile thermal probe connected to the ureteroscope was used to record temperature changes in the renal pelvis system as an outcome measure (Kose et al., 2024). Operation was performed using a high-power 100W holmium laser system (Lumenis VersaPulse^®^ PowerSuite™, Lumenis, Yokneam, Israel) with a 272 µm core diameter reusable optical fiber. The laser was operated in standard long-pulse mode (350–700 µs) to ensure efficient energy delivery. To monitor intra-luminal temperature, a 0.5-mm diameter flexible K-type thermocouple (calibrated ± 0.1 °C) was used. The probe was securely attached to the external sheath of the ureteroscope, with its tip positioned parallel to the laser fiber at a fixed lateral distance of one mm. During activation, the fiber-probe complex was maintained at a strictly controlled distance of one mm from the ureteral wall within the fluid-filled ureteral lumen, minimizing measurement variability caused by hand movements. To simulate a clinically rigorous lithotripsy scenario involving substantial stone burden, a dual-phase laser activation protocol was employed. The procedure commenced with an initial continuous activation for 30 s to mimic the rapid fragmentation of large calculi, followed by an intermittent duty cycle (5 s on, 5 s off) for the remaining duration to facilitate heat dissipation and visualization. The second project, investigating the ureteral repair process after perfusion with different temperatures of lavage fluid, involved dividing 6 piglets into groups (2 piglets per group), with the experimental ureters continuously perfused with 50 °C, 60 °C, and 70 °C hot lavage fluid for 20 min, respectively; ureteral tissue samples were collected on the day of surgery or 14 days postoperatively, fixed in 10% formalin, and embedded in paraffin. Masson staining was used to observe collagen fiber proliferation, with collagen tissue area quantitatively analyzed by randomly selecting three layers per ureter and three fields of view per layer, which served as key outcome indicators. The third project aimed to establish a ureteral stenosis model: after inserting the ureteroscope and confirming contralateral ureteral patency, a 30 W holmium laser was activated in the operated ureter to create an injury of approximately 10 mm in length (characterized by pale ureteral wall and surrounding mucosa, exposed muscle layer, no bleeding or perforation). Fourteen days postoperatively, ureteral stenosis was observed under a microscope to evaluate the relationship between injury extent and stenosis formation, which was a core outcome measure. Additionally, for all experimental projects, general conditions of the piglets (body weight, appetite, drinking water intake, activity status) were regularly monitored to assess health status and response to interventions. For renal tissue analysis in relevant projects, immunohistochemical staining was performed for inflammatory markers (NLRP3, IL-6, CD80) and fibrosis markers (COL-1, α-SMA, CD206); 5 fields of view at 20 × magnification were randomly selected for quantitative analysis of staining intensity, serving as outcome indicators for renal inflammation and fibrosis. Statistical analyses were conducted using two-way ANOVA with GraphPad Prism software (San Diego, CA, USA), with data presented as mean ± standard error and a *P*-value < 0.05 considered statistically significant.

### Staining procedure

Embed the tissue specimens in paraffin with a slice thickness of 4.5 μ m. The staining methods include Masson staining and immunohistochemical staining.

Masson staining is mainly used to display structures such as collagen fibers, mucus, and cartilage in tissues. The experimental steps include: dewaxing to water, staining the cell nucleus with Weigert hematoxylin or semen; afterwards, collagen fibers, mucus, and cartilage were treated with a series of specific staining solutions such as Masson’s acid counterstain, 2% glacial acetic acid aqueous solution, 1% phosphomolybdic acid aqueous solution, *etc*., resulting in a blue color for collagen fibers, mucus, and cartilage, while cytoplasm, muscle, *etc*., appeared red. After dehydration, transparency, and sealing, observe under a light microscope.

Immunohistochemical staining steps include routine dewaxing of sections in water, antigen repair, removal of endogenous peroxidase with 3% hydrogen peroxide solution, blocking, antibody incubation, DAB staining, hematoxylin counterstaining, dehydration, transparency, and sealing, followed by observation under a light microscope.

To ensure objectivity, histological scoring was performed by two independent pathologists who were blinded to the group allocation. Inter-observer reliability was confirmed using Cohen’s Kappa coefficient (*κ* = 0.85), indicating strong agreement between the two pathologists.

### Thermal dose calculation

To evaluate the cumulative biological effect of thermal exposure, the thermal dose was calculated as Cumulative Equivalent Minutes at 43 °C (CEM43) using the trapezoidal integration method, according to the formula proposed by Sapareto & Dewey (1984):

CEM = ∑R(43-T)Δt

where T is the average temperature during the time interval Δt, *R* = 0.5 for T > 4 °C, and *R* = 0.25 for T ≤ 43 °C. This metric allows for a standardized comparison of thermal injury risk across different temperature–time profiles.

### Statistical analysis

Data are shown as mean ± standard error. Statistical analyses were performed using the *t*-test and ANOVA. A *P*-value of less than 0.05 was considered significant. All statistical analyses were performed using the software PRISM (Graph Pad, San Diego, CA, USA).

**Figure 1:**
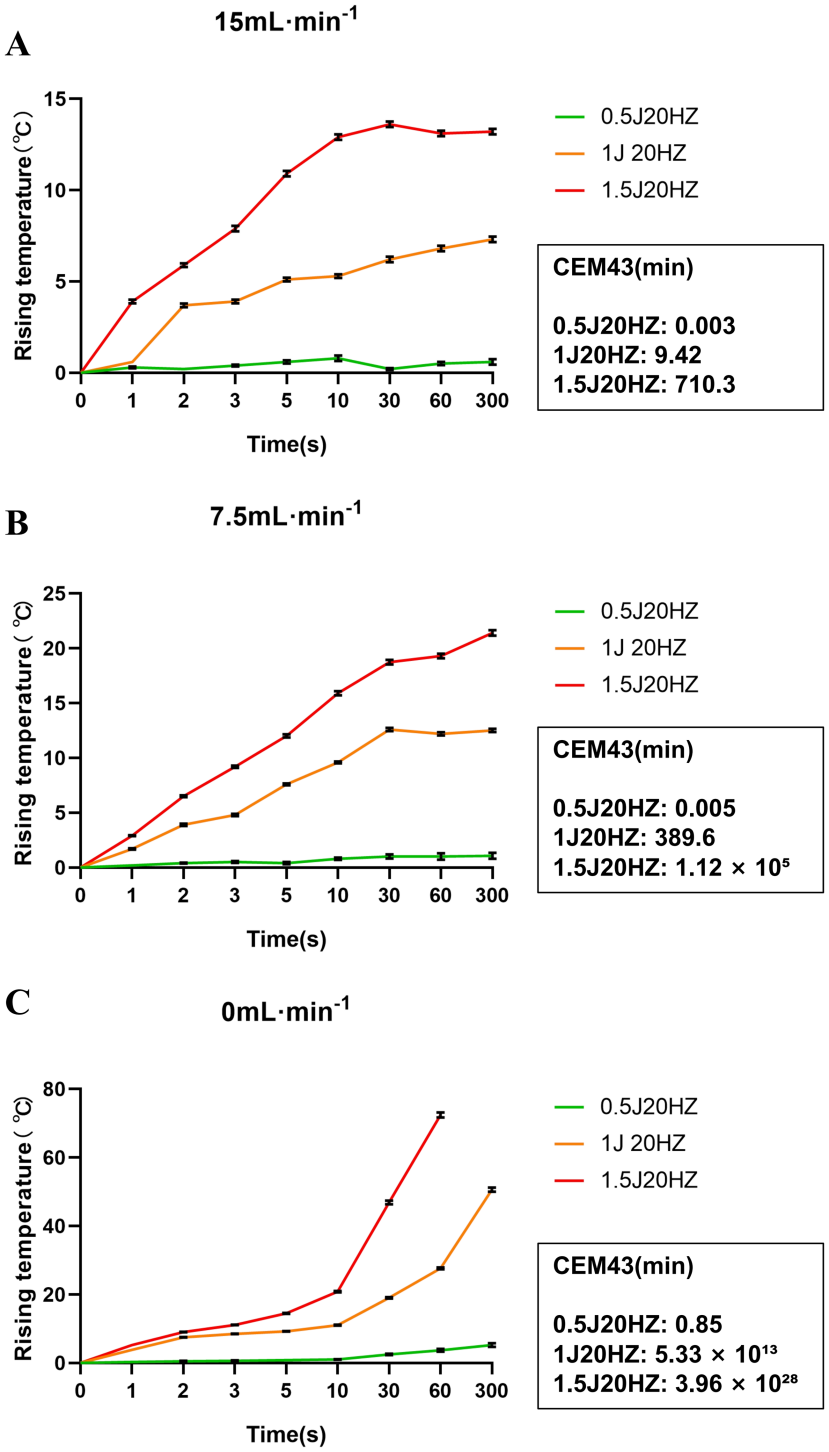
Time–temperature curves and thermal dose (CEM43) analysis under different holmium laser powers and lavage fluid flow rates. Data are presented as mean ± SEM (*n* = 3 piglets per group). Thermal dose was quantified using Cumulative Equivalent Minutes at 43° C (CEM43). (A) At a flow rate of 15 mL min^−1^, the temperature rise was minimal, with CEM43 values indicating negligible thermal injury risk (CEM43 < 1). (B) At 7.5 mL min^−1^, the temperature increased significantly with higher laser power. (C) In the absence of irrigation (0 mL min^−1^), the temperature rose rapidly to extreme levels. Note that the CEM43 values for 1.0 J and 1.5 J in this group exceeded 1,013, representing an upper-bound injury model rather than a standard clinical scenario.Statistical analysis was performed using two-way ANOVA. Data are presented as mean ± SEM, *n* = 3.

## Results

### The influence of lavage fluid flow rate and holmium laser power on the degree of lavage fluid temperature rise

In this experiment, three piglets were modeled to simulate ureteroscopic procedures. After general anesthesia, the piglets were placed in the supine position, and ureteroscopes were routinely inserted. After routine microscopic examination of the contralateral ureter for patency, the experimental side of the ureter was treated with different power, time and lavage fluid flow rates, respectively. We grouped the ureter with lavage fluid flow rates of 15 mL min^−1^, 7.5 mL min^−1^ and 0 mL min^−1^, respectively, and the degree of temperature rise caused by lasers of different powers varies (Fig. 1). When the lavage fluid flow rate was 15 mL min-1, the temperature rise remained below 13 °C even at 30 W. Correspondingly, the calculated thermal dose was negligible (CEM43 < 1), indicating a wide safety margin. At a flow rate of 7.5 mL min^−1^, the 30 W setting caused a temperature increase exceeding 20 °C, resulting in a significantly elevated thermal dose (CEM43 > 700 min), whereas 20 W resulted in a moderate rise (∼12 °C) with a much lower thermal dose (CEM43 ≈ 9.4 min). In the no-flow group (0 mL min^−1^), simulating an extreme failure of irrigation, the temperature rose rapidly. This group was designed to simulate clinical scenarios where irrigation is severely compromised, such as in cases of impacted stones or tight ureteral strictures where fluid outflow is obstructed. While 10W remained relatively safe (CEM43 ≈ 0.8 min), powers of 20 W and above caused the temperature to reach dangerous levels within seconds, yielding extreme CEM43 values (>10^13^). This confirms that without irrigation, high-power laser activation creates an immediate and catastrophic thermal injury risk.

### Repair process of living ureter after perfusion with different lavage fluid temperatures

**Figure 2:**
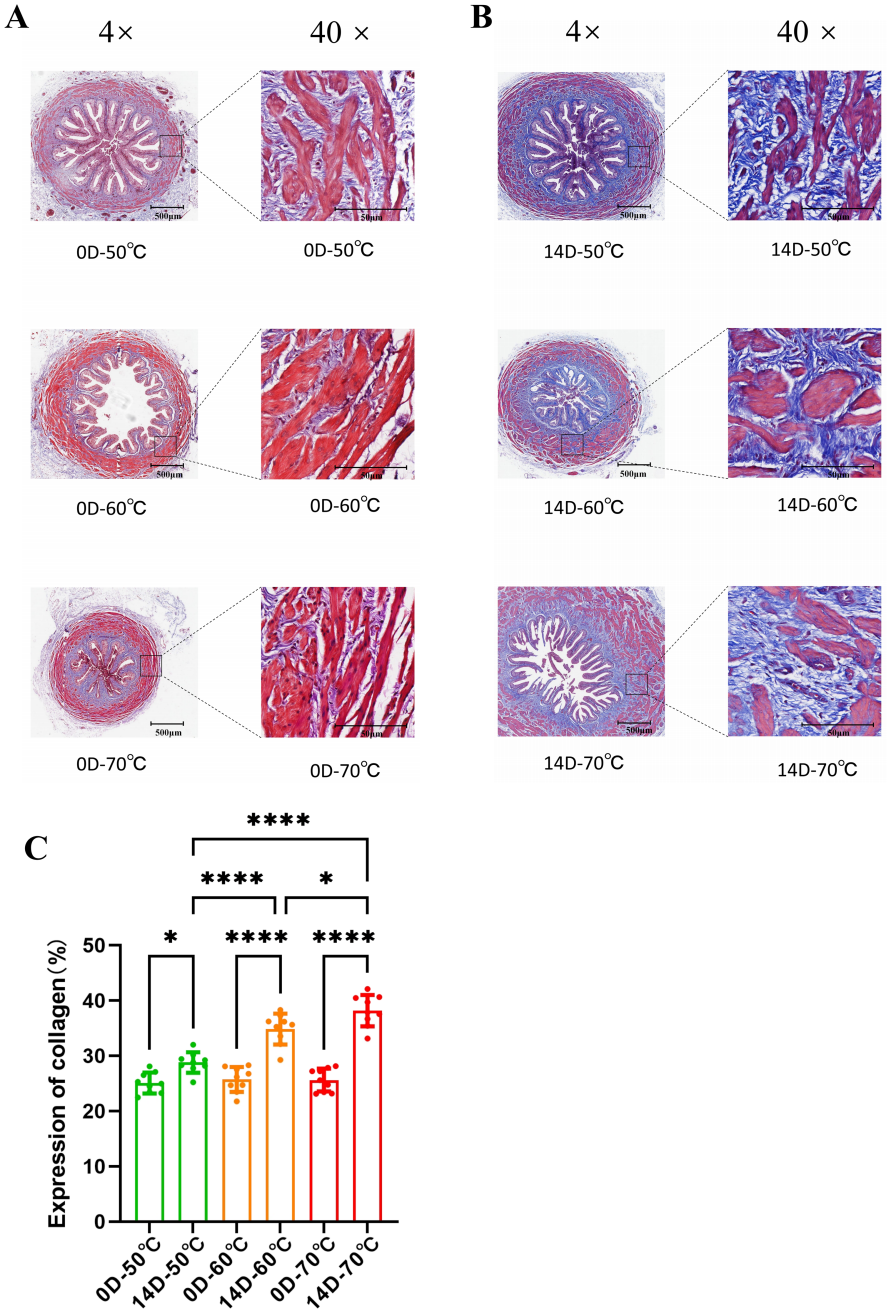
Histological evaluation of ureteral remodeling and collagen deposition following thermal exposure. The ureters were perfused with irrigation saline at 50 °C, 60 °C, and 70 °C for 20 min. (A) Masson’s trichrome staining of ureteral tissue on the day of surgery (Acute phase), observed at 4× and 40× magnification. (B) Masson’s trichrome staining at 14 days post-surgery (Repair phase), showing varying degrees of collagen thickening (blue staining). (C) Quantitative analysis of the collagen tissue area. There is a significant increase in collagen deposition on day 14 compared to the day of surgery, which correlates with the initial perfusion temperature. Statistical analysis was performed using two-way ANOVA. Data are presented as mean ± SEM , *n* = 9 fields of view; derived from 3 different sections × 3 randomly selected fields per ureter . *P < 0.05, **P < 0.01, ***P < 0.001, ****P < 0.0001.

Perform Masson staining on the collected ureteral sections. Observing the slices taken on the day of surgery, varying degrees of ureteral mucosal epithelial edema and epithelial cell shedding were observed in all ureters perfused with lavage fluid temperature. As time progressed, varying degrees of ureteral wall tissue damage, collagen tissue (blue area) proliferation, disordered muscle layer arrangement, and regeneration of shedding urinary tract epithelium were observed. At 14 days, partial neovascularization can be detected, and the ureteral wall is thicker than before (Figs. 2A, 2B). Randomly select three layers from each group of ureters, and then randomly select three fields of view on each layer. By statistically analyzing the collagen tissue area in these fields, it can be found that there is a significant difference in collagen tissue area between postoperative day and day 14 at the same temperature, and the proportion of collagen tissue area on day 14 after surgery also increases with the increase in perfusion temperature (Fig. 2C). This suggests that the repair process of thermal injury to our ureter is dominated by collagenous tissues.

### Inflammation and fibrosis degree of kidney at different times after holmium laser induced thermal injury

**Figure 3:**
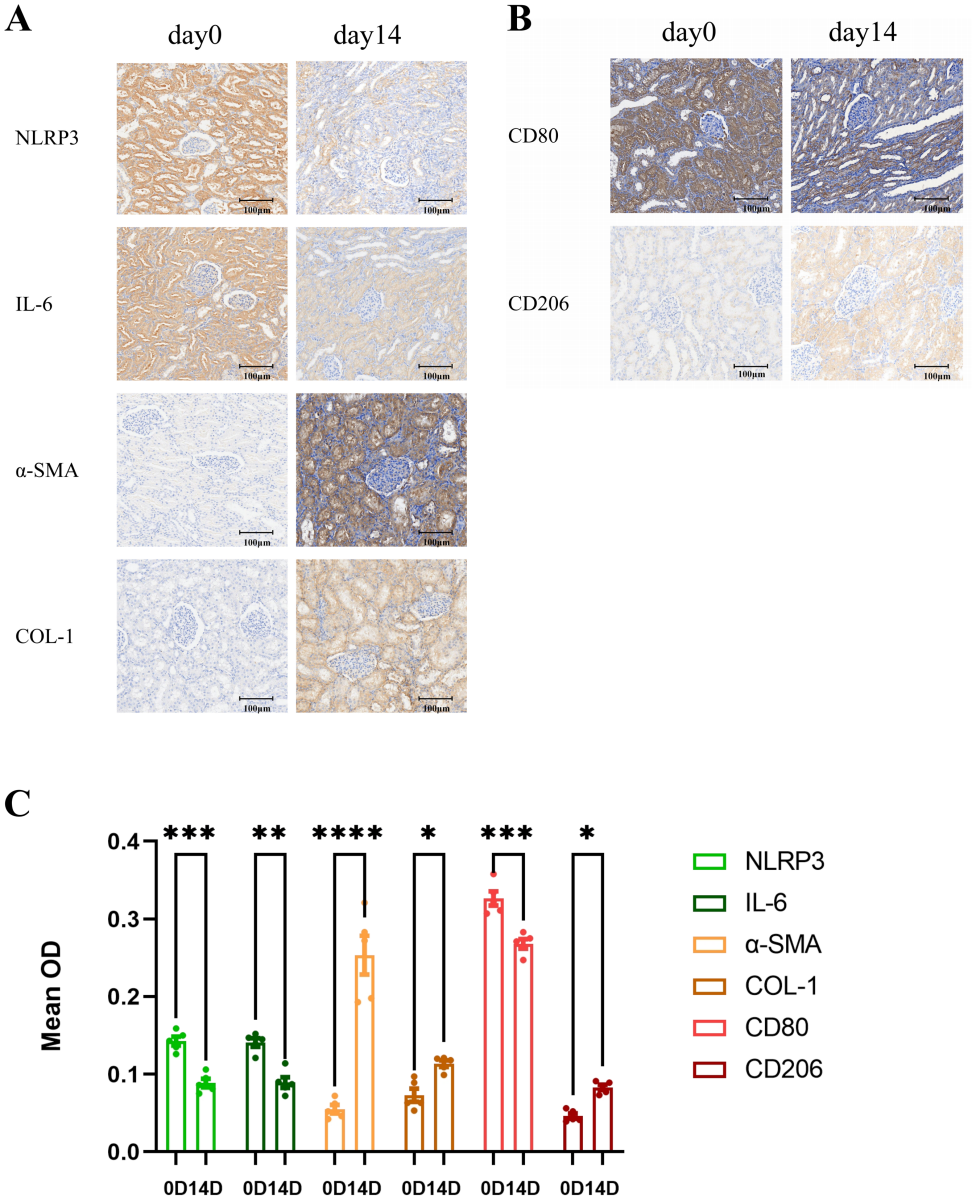
Dynamic changes in renal inflammation, fibrosis, and macrophage polarization following thermal injury. (A) Immunohistochemical (IHC) staining of renal tissue showing the transition from acute inflammation (NLRP3, IL-6) on Day 0 to fibrosis (COL-1, α-SMA) on Day 14 (20× magnification). (B) IHC staining illustrating the polarization of macrophages from the pro-inflammatory M1 phenotype (CD80 high) on Day 0 to the pro-reparative/fibrotic M2 phenotype (CD206 high) on Day 14 (20× magnification). (C) Quantitative analysis of the mean optical density (Mean OD) for all markers, confirming statistically significant differences between the acute and repair phases. Statistical analysis was performed using two-way ANOVA. Data are presented as mean ± SEM, *n* = 5 randomly selected fields of view. *P < 0.05, **P < 0.01, ***P < 0.001, ****P < 0.0001.

The kidney tissue of piglets receiving 70 °C lavage solution was divided into two groups on the day of surgery and 14 days after surgery. Immunohistochemical staining was performed, randomly selecting 5 fields of view at a magnification of 20 × and quantitatively analyzing their immunohistochemical staining intensity. Under a microscope, mild scar formation can be observed in the damaged kidney. This is associated with varying degrees of tubular dilation, flattening of tubular epithelial cells, and relative compression of the glomeruli. Increased fluid in the renal interstitial, mild edema, and infiltration of inflammatory cells. Immunohistochemical staining was performed on the inflammatory markers NLRP3 and IL-6. It was found that they were significantly upregulated on the day of surgery, while weakly expressed on the 14th day after surgery. The trend of fibrosis indicators COL-1 and α- SMA is opposite to that of inflammation indicators, with almost no expression on the day of surgery, but significantly high expression on day 14 after surgery (Fig. 3A). This suggests that the damage to the kidneys caused by high lavage fluid temperature may be a trend of gradually decreasing inflammation levels and increasing fibrosis levels. To verify this viewpoint, we performed immunohistochemical staining on M1 macrophage marker CD80 and M2 macrophage marker CD206 separately. We found that CD80 was highly expressed on the day of surgery and decreased on day 14 after surgery, while CD206 was lowly expressed on the day of surgery and increased on day 14 after surgery (Fig. 3B). This suggests that during the repair process of kidney thermal injury, macrophages transform from M1 type to M2 type. There were statistically significant differences in the immunohistochemical staining intensity of all the above markers on the day of surgery and 14 days after surgery (Fig. 3C).

### Relationship between the extent of holmium laser injury and ureteral stenosis

**Figure 4:**
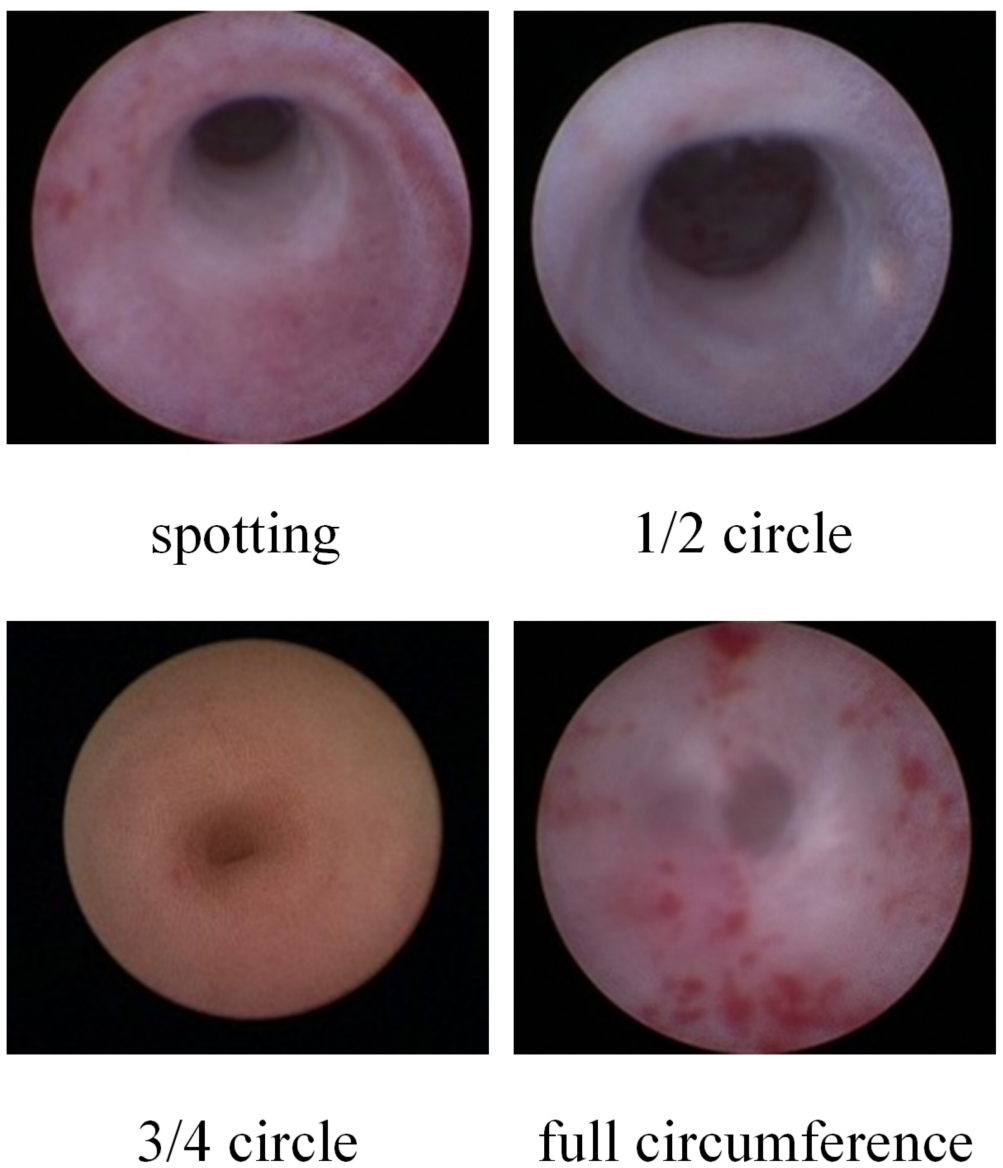
Endoscopic visualization of ureteral stricture formation relative to the circumferential extent of laser injury. Representative ureteroscopic images taken 14 days post-surgery. Injuries involving “spotting” or “1/2 circle” of the ureteral circumference showed good healing without obstruction. In contrast, anatomically significant ureteral stricture was observed only when the laser-induced injury involved ≥3/4 of the circumference or the full circumference.

In the aforementioned experiments, even though the lavage fluid temperature exceeded the safe range, there was still no significant ureteral stricture. To clarify the relationship between ureteral injury and the extent of ureteral stenosis, we established models in three piglets. Different ranges of holmium laser damage to the ureteral wall were used respectively, and the different ranges included spotting, 1/2 circle, 3/4 circle, and full circumference. The holmium laser power was 1.5J/20HZ, the lavage fluid flow rate was 7.5 mL min^−1^, and the holmium laser was stimulated at a distance of about one mm from the ureteral wall. The degree of injury was that the holmium laser invaded the muscularis propria but did not break through it. Fourteen days later, under the microscope, the ureters of the piglets with punctate and 1/2 circle injuries showed no obvious ureteral stenosis and smooth ureteroscopy insertion, although a little scar formation was seen. In contrast, obvious ureteral stenosis formation was seen in ureters with 3/4 circle and full circumference injuries (Fig. 4).

## Discussion

Medically induced ureteral stricture is a serious complication after a ureteroscopy (Moretto et al., 2024). If untreated, it may lead to serious consequences such as hydronephrosis and decreased renal function. A number of factors have been suggested to be potentially related to the formation of ureteral strictures, such as excitation of the ureteral wall by the laser, friction between the mirror and the ureteral wall, and elevation of the lavage fluid temperature due to laser energy. Although the cause of medically induced ureteral stenosis is not fully understood, clinicians are generally wary of, or even intimidated by, this complication. Defining the cause of medical ureteral stenosis is useful not only to help clinicians avoid complications, but also to help clinicians remain calm and avoid an overly stressful state of mind when performing the necessary medical procedures.

Many previous articles have reported the occurrence of ureteral stricture after ureteroscopy, and thermal injury is believed to be a key factor contributing to this phenomenon. Temperatures exceeding 43 °C are considered dangerous (Tonyali et al., 2023a; Tonyali et al., 2023b; Rice et al., 2022; Liang et al., 2020). Some scholars also believe that 54 °C is the threshold for causing thermal damage (Peteinaris et al., 2022). In previous studies, laser lithotripsy was performed on isolated pig kidneys using conventional lithotripsy power. The highest intraoperative temperature did not exceed 50 °C. At this temperature, there is almost no damage to the renal tubules, only mild damage to the urinary tract epithelium and subcutaneous tissue. The relationship between factors such as holmium laser excitation time and power and temperature increase in *in vitro* experiments has also been reported (Rezakahn et al., 2022; Louters et al., 2022; Sourial et al., 2018), but evidence from *in vivo* experiments is lacking (Teng et al., 2021). We carried out the first *in-vivo* experiments to simulate this process. The results showed that in the presence of 15 mL min^−1^ aqueous perfusion, an increase in lavage fluid temperature to the point of ureteral injury was almost impossible to achieve. Our observations align closely with the thermal safety boundaries proposed by Marom et al. (2023), who established that an irrigation flow rate of at least 15 mL min^−^^1^ is critical to maintain thermal safety at moderate-to-high power settings (*e.g.*, 25 W). Consistent with their *in vivo* safety maps, we found that reducing the flow rate to 7.5 mL min^−^^1^ at 30 W resulted in a rapid accumulation of thermal dose, confirming that insufficient irrigation is a primary determinant of thermal injury risk. This mechanism is further supported by Bonzagni et al. (2025) whose *in vivo* data demonstrated that even with high-power settings, thermal injury can be mitigated when strict irrigation protocols and intermittent activation strategies are adhered to. While low-power lasers are still considered safe despite the absence of lavage fluid flow perfusion, at high power it takes only a few seconds to raise the lavage fluid temperature to dangerous temperatures. The possibility of the discrepancy between the results of the present study and previous studies is that in the *in vivo* situation, the ureter is adjacent to many tissues in the abdominal cavity and is subjected to the thermal effects produced by holmium laser excitation as soon as possible, but good thermal conductivity results in a gentle rise in temperature. In ex vivo experiments, the isolated ureter is surrounded by air, which is a poor conductor of heat and has very poor heat dissipation efficiency and cannot simulate the actual situation in the abdominal cavity. Moreover, there is an intrinsic thermoregulatory mechanism in living animals (Nakamura, 2024). There have been studies showing that in the presence of blood flow perfusion, the temperature rise caused by holmium laser leads to faster cooling when returning to normal temperature levels (Marom et al., 2024). This may be the reason for the differences between this experiment and many previous *in vitro* experiments. So our *in vivo* experiments have significantly higher credibility than previous experiments. Furthermore, while our study utilized a high-power Holmium:YAG (Ho:YAG) system, we acknowledge the evolving landscape of laser technology. A recent comparative study (Tang et al., 2024) highlighted differences in heat generation profiles between Ho:YAG and the emerging Thulium Fiber Laser (TFL). Although TFL may offer different ablation efficiencies, the fundamental principles of thermodynamics observed in our study—specifically the dependence of mucosal safety on irrigation flow rate and operation mode—remain universally applicable across laser platforms.

When we use lavage fluids of different temperatures during surgery, the ureter undergoes a series of morphological changes during the injury and repair process. We perfused the ureter with lavage fluid flow at three temperature gradients of 50 °C, 60 °C, and 70 °C for 20 min until 14 days after surgery. There was still no bleeding or ureteral stenosis. In ureteroscopic surgery, postoperative ureteral stricture may be caused by various reasons, such as direct laser irradiation, elevated lavage fluid temperature, friction between the endoscope and the ureteral wall, *etc* (Darwish et al., 2019). Thermal effects are not the cause of ureteral stricture. The results of this experiment show that the thermal effect generated by holmium laser can cause a certain degree of damage to ureteral tissue and kidney tissue, but not to the extent of causing ureteral stenosis.

In this experiment, we investigated the repair process of ureteral injury using Masson trichrome staining. The results showed that as time progressed, the main structural changes in the ureter were collagen thickening. In contrast, the arrangement of the ureteral muscle layer tended to be chaotic. At the same time, the ureteral wall thickened significantly, and new capillaries began to appear on day 14. The shedding urinary tract epithelium regenerated without being covered by other tissues or undergoing metaplasia. In addition, the degree of ureteral injury tends to be more severe due to the increase in lavage fluid temperature. This experiment demonstrates that during the repair process of ureteral thermal injury, collagen tissue and connective tissue proliferation are the main factors. Although there is no obvious ureteral stenosis, the ureter becomes stiff and has reduced extensibility. This is due to collagen and connective tissue proliferation. Along with structural changes, there may also be a certain degree of inhibition in electrophysiological processes such as signal transduction in the ureter (Hammad et al., 2011). This may be a common cause of mild hydronephrosis and fibrosis in damaged kidneys.

Through immunohistochemical staining of multiple indicators, we have provided an overview of the repair process of live pig kidneys after thermal injury. The experimental results showed that the repair process of kidneys and ureters after thermal injury is a parallel process of gradually decreasing inflammatory response and gradually increasing fibrosis level. Inflammatory indicators such as NLRP3 and IL-6 were significantly overexpressed on the day of surgery, but weakly expressed on the 14th day after surgery; Fibrosis indicators such as COL-1 and α- SMA are the opposite. In addition, through immunohistochemical staining of macrophage markers CD80 and CD206, there is evidence to suggest that macrophages are closely associated with the repair process of kidneys after holmium laser thermal injury. The results showed that CD80 expression levels gradually decreased with time, while CD206 expression levels gradually increased with time. M1 macrophages have pro-inflammatory effects, clear infections, and promote kidney damage, while M2 macrophages have reparative effects and play an critical role in the progression of kidney fibrosis (Lu et al., 2018).

There are some differences in the repair process of the kidney and ureter depending on the type of damage sustained. Reports have shown that when ureteral stents cause ureteral stenosis, there is a tendency for the level of inflammation to increase in parallel with the level of fibrosis (Reicherz et al., 2023), which is different from the present study. Although both high lavage fluid temperature and ureteral stenting damage the ureteral wall, the level of inflammation that progresses over time does not show the same trend. This may be due to differences between these two injury processes. This may be due to the fact that hyperthermia is a transient factor that leads to acute inflammation. After the risk factors are removed, the level of inflammation gradually decreases, the ureteral epithelium begins to repair, and the level of fibrosis continues to rise. Ureteral stents, on the other hand, are continually placed foreign bodies that cause a chronic inflammatory response to constant ureteral irritation. This chronic inflammation is accompanied by a rising level of fibrosis.

**Figure 5:**
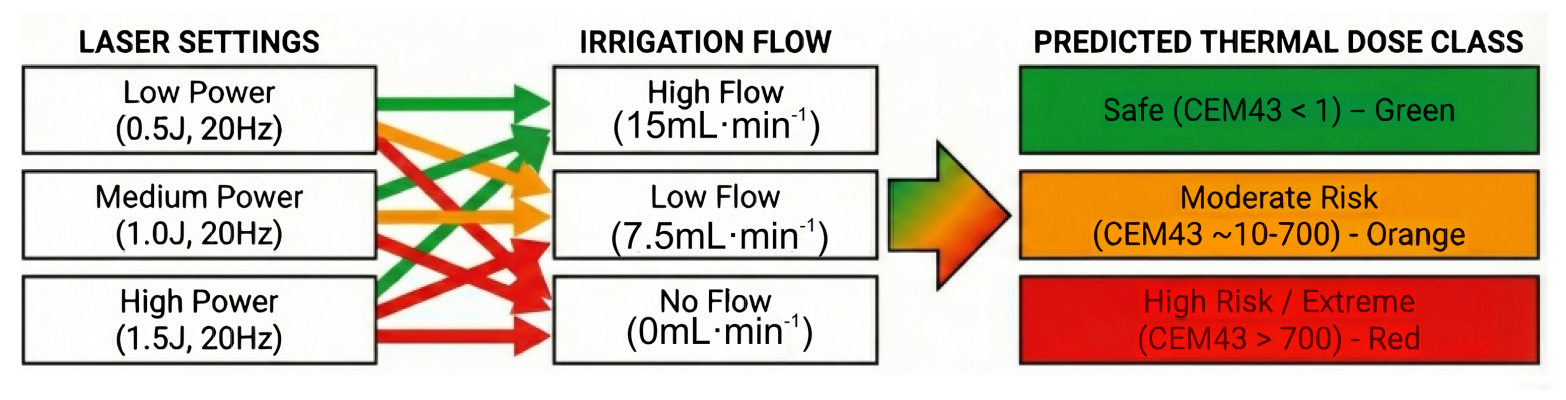
Clinical decision flowchart summarizing the relationship between laser settings, irrigation flow rate, and thermal injury risk. The flowchart illustrates that at Low Power settings (*e.g.*, 10 W), thermal risk is generally managed by adequate irrigation. However, at High Power settings (*e.g.*, ≥ 20 W), maintaining a High Flow rate ≥15 mL min^−1^) is critical to remain in the Safe zone (green box). The combination of High Power and Low Flow (<15 mL min^−1^) leads to the highest thermal dose accumulation, resulting in a High Risk of extensive thermal injury and subsequent ureteral stricture formation (red box).

Ureteral injury is closely associated with ureteral stricture (Tonyali et al., 2023b), but not all minor injuries can cause ureteral stricture. Currently, there is no research exploring the relationship between the extent of ureteral injury and the formation of ureteral stricture. This was modeled in our experiments, and anatomical ureteral stenosis did not occur until holmium laser injury reached more than 3/4 of a circle. When the ureter is damaged at a point or 1/2 turn, although ureteral motility and electrophysiologic activity may be reduced and excretory function may be impaired as a result, anatomic ureteral stenosis does not occur and ureteroscopy can still be inserted without difficulty. In clinical practice, when the holmium laser damages the ureter to 3/4 circle or more, the patient should be alerted to postoperative ureteral stenosis, and appropriate measures should be taken at an early stage. To assist urologists in optimizing intraoperative safety, a clinical decision flowchart summarizing the relationship between laser settings, irrigation flow, and thermal risk is presented in Fig. 5.

There are still many shortcomings in this experiment. First, the sample size of this experiment is limited, which restricts the experiment’s progress and the generalization of conclusions. Second, regarding the observation period, we acknowledge that 14 days represents the acute-to-subacute phase of injury. While this timeframe effectively captured the critical transition from inflammation to early fibrosis (as evidenced by increased collagen deposition), the formation of mature, functionally obstructive strictures may evolve over months. Future long-term survival studies are warranted to determine whether the mechanical injury threshold identified here (≥3/4 circumference) invariably progresses to permanent stenosis. Third, distinct inter-species differences must be acknowledged when extrapolating these findings to clinical practice. Although the porcine ureter is the standard endourological model due to its anatomical resemblance to humans, its urothelium and muscularis layers are generally thicker, potentially offering greater intrinsic thermal resilience than human tissue. Furthermore, our study utilized healthy ureters with intact vascular perfusion. In clinical scenarios, patients often present with chronic inflammation or impacted stones, where local edema or compromised blood flow could impair the natural “heat sink” effect, making the tissue more susceptible to thermal injury. Therefore, the CEM43 thresholds identified here should be viewed as baseline safety reference values rather than absolute clinical limits.

## Conclusion

Reasonable control of the power and intraoperative perfusion flow rate of holmium lasers can effectively avoid water temperature exceeding the safe range during ureteroscopy surgery. The short-term high temperature caused by holmium lasers is not the cause of ureteral stricture. When repairing thermal injury in the ureter and kidneys, collagen tissue proliferation was observed concurrently with a decrease in inflammation levels and an increase in fibrosis levels over time. When the holmium laser causes ureteral injury that invades the muscle layer for 3/4 circle or more, it will lead to visible ureteral stenosis under endoscopy.

## Supplemental Information

10.7717/peerj.21015/supp-1 Supplemental Information 1 The data of lavage fluid flow rate and holmium laser power on the degree of lavage fluid temperature rise We grouped the ureter with lavage fluid flow rates of 15 ml/min, 7.5 ml/min and 0 ml/min respectively, and the degree of temperature rise caused by lasers of different powers varies. Here is the raw data regarding the temperature increase.

10.7717/peerj.21015/supp-2 Supplemental Information 2 Raw data for Fig. 2C Percentage of collagen in Masson staining

10.7717/peerj.21015/supp-3 Supplemental Information 3 Raw data for Fig. 3C Mean OD value of immunohistochemistry

10.7717/peerj.21015/supp-4 Supplemental Information 4 ARRIVE 2.0 checklist

## Additional Information and Declarations

### Data Availability

The following information was supplied regarding data availability: The raw measurements are available in the Supplemental Files.
